# Imaging fictive locomotor patterns in larval *Drosophila*

**DOI:** 10.1152/jn.00731.2015

**Published:** 2015-08-26

**Authors:** Stefan R. Pulver, Timothy G. Bayley, Adam L. Taylor, Jimena Berni, Michael Bate, Berthold Hedwig

**Affiliations:** ^1^School of Psychology and Neuroscience, University of St Andrews, St Andrews, United Kingdom;; ^2^Department of Zoology, University of Cambridge, Cambridge, United Kingdom; and; ^3^Janelia Research Campus, Howard Hughes Medical Institute, Ashburn, Virginia

**Keywords:** calcium imaging, central pattern generator, intersegmental coordination, locomotion, neuroethology

## Abstract

We have established a preparation in larval *Drosophila* to monitor fictive locomotion simultaneously across abdominal and thoracic segments of the isolated CNS with genetically encoded Ca^2+^ indicators. The Ca^2+^ signals closely followed spiking activity measured electrophysiologically in nerve roots. Three motor patterns are analyzed. Two comprise waves of Ca^2+^ signals that progress along the longitudinal body axis in a posterior-to-anterior or anterior-to-posterior direction. These waves had statistically indistinguishable intersegmental phase delays compared with segmental contractions during forward and backward crawling behavior, despite being ∼10 times slower. During these waves, motor neurons of the dorsal longitudinal and transverse muscles were active in the same order as the muscle groups are recruited during crawling behavior. A third fictive motor pattern exhibits a left-right asymmetry across segments and bears similarities with turning behavior in intact larvae, occurring equally frequently and involving asymmetry in the same segments. Ablation of the segments in which forward and backward waves of Ca^2+^ signals were normally initiated did not eliminate production of Ca^2+^ waves. When the brain and subesophageal ganglion (SOG) were removed, the remaining ganglia retained the ability to produce both forward and backward waves of motor activity, although the speed and frequency of waves changed. Bilateral asymmetry of activity was reduced when the brain was removed and abolished when the SOG was removed. This work paves the way to studying the neural and genetic underpinnings of segmentally coordinated motor pattern generation in *Drosophila* with imaging techniques.

central pattern generating (CPG) networks produce coordinated motor output without sensory feedback and underlie many behaviors, such as walking, flying, singing, and eating (for reviews see Delcomyn 1980; Marder and Calabrese 1996; Mulloney and Smarandache 2010). Interrogation of CPG networks often relies upon preparations that produce “fictive” motor patterns, which resemble patterns of neuronal activity in intact animals but are produced in the absence of sensory feedback. The use of such preparations has helped to uncover general principles of how CPG networks function at the level of identified neurons and synapses (for reviews see Ayali and Lange 2010; Marder and Calabrese 1996; Mulloney and Smarandache-Wellmann 2012). Recently, optical imaging of neural activity in these preparations has allowed the interaction between network components to be monitored in real time (Briggman and Kristan 2006; Kwan et al. 2010; Warp et al. 2012). However, a fundamental aspect of many behaviors, such as swimming (Hocker et al. 2000), crawling (Puhl and Mesce 2008), and ventilation (Lewis et al. 1973; Ramirez and Pearson 1989), is the coordination between multiple body segments, and in many species studying these networks with imaging techniques is difficult because of the large distances between the neurons involved.

To overcome these limitations, we have studied the larval locomotor system of *Drosophila*. The *Drosophila* nervous system is relatively complex, consisting of 10,000–15,000 neurons (Iyengar et al. 2006; Truman et al. 1993), and produces a repertoire of segmentally coordinated locomotor behaviors including crawling, turning, rolling, and burrowing (Berni et al. 2012; Fox et al. 2006; Gomez-Marin et al. 2012; Hwang et al. 2007; Saraswati et al. 2004). Unlike most arthropods, the larval central nervous system (CNS) is highly compact, as the abdominal and thoracic ganglia are fused (Niven et al. 2008), making it well suited for functional imaging. Critically, the system is also genetically manipulable, allowing the expression of transgenes that facilitate imaging and manipulation of neuronal activity in specific subsets of neurons.

Progress has recently been made in understanding how the larval *Drosophila* locomotor system functions, from detailed descriptions of muscle coordination during crawling (Gjorgjieva et al. 2013; Heckscher et al. 2012) to analyzing how parts of the nervous system contribute to the coordination and speed of locomotion, including the brain (Berni et al. 2012), sensory neurons (Caldwell et al. 2003; Hughes and Thomas 2007; Song et al. 2007), motor neurons (Inada et al. 2011), and subsets of interneurons (Kohsaka et al. 2014; Okusawa et al. 2014). CPG networks are likely to underlie at least some larval behaviors, because using genetic tools to inhibit synaptic release in sensory neurons does not abolish crawling behavior in larvae (Hughes and Thomas 2007; Suster and Bate 2002). Also, when all sensory feedback is surgically removed, the CNS endogenously produces segmentally coordinated motor output (Berni 2015; Fox et al. 2006). However, the nature and coordination of this endogenous motor activity have not yet been systematically investigated.

We have used a genetically encoded Ca^2+^ indicator (GCaMP3; Tian et al. 2009) to image motor activity simultaneously across thoracic and abdominal ganglia in the larval CNS. We combine this with electrophysiology and behavioral analysis to demonstrate that the isolated larval CNS produces a range of motor patterns that are quantifiably similar to crawling behavior in intact larvae. By sequential ablation of sections of the CNS, we explore how regions of the CNS contribute to the patterns. This work provides a foundation for future work on the neural basis of crawling in *Drosophila* larvae using imaging techniques and opens up new possibilities for the study of intersegmental coordination in a genetically manipulable organism.

## MATERIALS AND METHODS

### 

#### Animal rearing and genetic constructs.

Fly larvae were reared on cornmeal-based food or sucrose-enriched agar and dried baker's yeast or on yeast alone. For imaging experiments, we used the GAL4-UAS system (Brand and Perrimon 1993) to drive expression of the Ca^2+^ indicator GCaMP3 (Tian et al. 2009). OK371-GAL4 (Mahr and Aberle 2006) was used for expression in all glutamatergic neurons, including all motor neurons; RRAF-GAL4 (Worrell and Levine 2008) for a motor neuron (aCC) that innervates a dorsal longitudinal muscle (DO1); BAR-GAL4 (Garces et al. 2006) for a group of motor neurons (LT MNs) that innervate lateral transverse muscles (LT 1–4); and RN2-Flp, Tub-FRT-CD2-FRT-GAL4 (Landgraf et al. 2003) for two motor neurons (aCC and RP2), to identify their projection through nerve roots. We used both Oregon R (OrR) and OK371-GAL4 × UAS-GCaMP3 (OK371-GCaMP) larvae in behavioral experiments.

#### Isolated CNS dissection.

For imaging and electrophysiology experiments, individual third instar larvae were positioned dorsal side up on Sylgard-lined petri dishes and pinned through the mouthparts and posterior abdomen. An incision along the dorsal surface was made with fine scissors, and the internal organs were removed. The body wall was then pinned flat. The CNS, including the brain, subesophageal ganglion (SOG), and ventral nerve cord (VNC), was dissected away from the larval body wall and positioned dorsal side up, secured at segmental nerves by pins fashioned from fine tungsten wire (California Fine Wire, Grover Beach, CA). In a subset of experiments, we positioned the isolated CNS on a coverslip coated in poly-d-lysine and then used either fine scissors or 18-gauge syringe tip needles to remove sections of the nervous system. Recordings were made at least 15 min after ablation. For all dissections and experiments, the CNS was covered with physiological saline containing (in mM) 135 NaCl, 5 KCl, 2 CaCl_2_, 4 MgCl_2_, 5 TES, and 36 sucrose.

#### Electrophysiology.

In *Drosophila* embryos, the axons of motor neurons innervating the lateral transverse and dorsal longitudinal muscles run in different nerves (intersegmental and segmental nerve, respectively). However, in third instar larvae, both nerves are bundled together; we refer to this bundle as the common nerve root (CNR). We used suction electrodes to measure activity in CNRs containing motor neurons that, prior to severing, project to the muscle field of individual abdominal hemisegments. Borosilicate glass capillaries were pulled with a P90 electrode puller (Sutter Instruments, Novato, CA), and tapered tips were broken to produce suction electrodes that fit tightly around single nerve roots. Electrodes were maneuvered with a MP-285 (Sutter Instruments) or a Leitz micromanipulator (Leica Microsystems, Wetzlar, Germany). Electrophysiological signals were amplified with a model 1700 extracellular amplifier (A-M Systems, Sequim, WA) and recorded with a PowerLab 16/30 data acquisition system and LabChart 7.1 software (AD Instruments, Colorado Springs, CO). Recordings were analyzed off-line with custom scripts in MATLAB (MathWorks, Natick, MA) and Spike2 (Cambridge Electronic Design, Cambridge, UK).

#### Live imaging in isolated CNS.

**Fig. 1.**
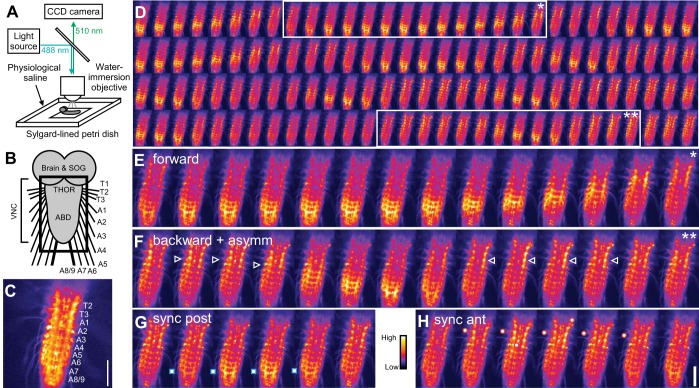
Ca^2+^ signals in the isolated central nervous system (CNS) of larval *Drosophila*. *A*: experimental setup. *B*: diagram of CNS. Black square indicates region imaged in following panels. SOG, subesophageal ganglion; THOR, thorax; ABD, abdomen; VNC, ventral nerve cord. *C*: still image of Ca^2+^ signal. CNS segments labeled from thoracic segment 2 (T2) to fused abdominal segment A8/9. *D*: sequence of still images of Ca^2+^ signals, running from *top left* to *bottom right*; successive images are 1.9 s apart. Posterior-to-anterior (forward, *) and anterior-to-posterior (backward, **) Ca^2+^ waves are shown. *E* and *F*: magnified view of forward wave (*E*) and backward wave (*F*), also showing bilaterally asymmetric activity in thoracic and anterior abdominal segments (white arrowheads). *G* and *H*: synchronous activity in posterior (*G*) and anterior (*H*) segments. Raw fluorescence intensity is indicated by color map scale bar. GAL4 Driver: OK371-GAL4. Scale bars, 100 μm.

We used wide-field epifluorescence microscopy for all live imaging experiments (Fig. 1*A*). An Optoscan monochromator (Cairn Research, Faversham, UK) uniformly illuminated the preparations with 488 ± 15-nm light using either a Leica DM-LFS (Leica Microsystems) or an Olympus X50WI compound microscope (Olympus, Center Valley, PA). Emitted light passed through standard green fluorescent protein (GFP) emission filters before reaching an Andor DU897 EMCCD camera (Andor Technologies, Belfast, UK). Images were captured at 5 or 10 Hz with Andor IQ software and constant gain settings. Optical and electrical recordings were synchronized with pulses generated during each camera exposure. Images were stabilized against lateral shifts in ImageJ (NIH, Bethesda, MD). Fluorescence values were extracted from regions of interest (ROIs) in thoracic (T2–T3) and abdominal (A1–A8/9) ganglia with ImageJ or custom MATLAB scripts. T1 and the SOG were obscured by the brain and were not analyzed. Extracted optical signals were analyzed in ImageJ, MATLAB, and Spike2. Signals are expressed as the percent change in fluorescence from baseline, ΔF/F. Changes of 50% ΔF/F were typical in all segments.

#### Confocal microscopy.

In a subset of experiments, we fixed larval CNSs in 4% paraformaldehyde and then imaged GFP fluorescence with a TCS-SP-5 confocal microscope (Leica) using LAS AF software as described previously (Berni et al. 2012).

#### Larval crawling behavior.

For behavior experiments, third instar larvae were washed and transferred to a petri dish thinly coated with 0.8% agarose. To examine the basic dynamics of crawling, we placed single larvae into a 23 × 23-cm square acrylic tray and, after 30 s of acclimatization, imaged larval behavior for 4–5 min. Images were captured at 26 Hz with a Stingray CCD camera (Allied Vision Technologies, Stadtroda, Germany) mounted on a Leica M165 FC dissecting microscope. In a separate set of experiments, we used higher magnification to analyze the kinematics of body wall movements in freely crawling animals. In these experiments, animals were placed in a smaller (10-cm diameter) petri dish, which was inverted to facilitate imaging of the ventral cuticle and denticle bands. Denticle bands are rows of hairs located near abdominal segmental boundaries and the attachment sites for dorsal and ventral muscle groups. These images were captured at 30 Hz with a TK0C1380 camera (JVC, Yokohama, Japan) mounted on a dissecting microscope. Data were recorded with a DSR-30P video recorder (Sony, Tokyo, Japan). After 30-s acclimatization, larvae were imaged for 1–2 min. Because crawling larvae rarely produced more than one or two backward waves at a time, in some experiments we promoted backward crawling by tying a filament of dental floss around the thorax. This treatment successfully induced bouts of 3–10 backward waves.

As in previous studies (Berni 2015; Berni et al. 2012; Gjorgjieva et al. 2013), we measured the distance between denticle bands in adjacent segments to analyze intersegmental coordination in crawling larvae. Denticle bands are pigmented and easily visible from segments A1–A8, and therefore data for contraction of segments can be calculated for A1–A7. Denticle movements were tracked with the “Manual Tracking” plug-in for ImageJ. Data were processed in Excel (Microsoft, Redmond, WA) and further analyzed in Spike2 and MATLAB.

#### Coherence-based analysis of periodic activity.

In a subset of experiments, we determined the phase relationships between segments for both imaging and behavioral data, using direct multitaper estimates of power spectra and coherence (Cacciatore et al. 1999; Percival and Walden 1993; Taylor et al. 2003). In all experiments, we determined the dominant frequency of waves in segment A7 from the power spectrum and calculated the coherence with other segments at that frequency (Thompson and Chave 1991). Time delays were sometimes calculated by multiplying the phase value by the dominant frequency. Coherence is a complex-valued quantity, comprising both a phase value and a magnitude. Standard statistical tests were used to determine whether the coherence magnitude was significantly different from zero (Jarvis and Mitra 2001). To calculate 95% confidence intervals we used a jackknife technique (Thompson and Chave 1991). Spectral calculations were carried out with custom scripts written in MATLAB.

#### Statistics.

All values are given as means ± SD unless otherwise stated. We tested data for normality using the Shapiro-Wilk test, with α = 0.05. When data were normally distributed, *t*-tests were used to test for significant differences or analysis of variance (ANOVA) followed by Tukey-Kramer post hoc analysis for multiple comparisons. For data that were not normally distributed, two-sample Wilcoxon tests were used or Kruskal-Wallis tests followed by Neuman-Keuls post hoc analysis for multiple comparisons. Statistical analyses were carried out in Excel, R (R Foundation, Vienna, Austria), SigmaPlot (Systat, San Jose, CA), and MATLAB.

## RESULTS

To characterize motor output in the isolated CNS optically, we expressed GCaMP3 in all motor neurons with a glutamatergic neuron-specific GAL4 driver, OK371, and imaged the CNS with epifluorescence microscopy (Fig. 1, *A–C*).

### 

#### Characterization of Ca^2+^ signals in the isolated CNS.

The CNS regularly produced three stereotyped patterns of motor activity endogenously, without electrical or pharmacological manipulation (Fig. 1, *D–F*; Supplemental Movie S1).^1^ Two were visible as bilaterally symmetric metachronal waves of Ca^2+^ fluorescence, in either a posterior-to-anterior (Fig. 1*E*) or an anterior-to-posterior (Fig. 1*F*) direction. The order of recruitment of segments during these waves was similar to forward and backward locomotion during crawling behavior, so we define these as “forward” and “backward” Ca^2+^ waves, respectively. A third pattern was bilaterally asymmetric activity (Fig. 1*F*; Supplemental Movie S2), which was frequently concomitant with backward waves (19 ± 7% of asymmetric activity; *N* = 10) and occasionally with forward waves (2.6 ± 2.5% of asymmetric activity; *N* = 10). Forward and backward waves showed synchronous activity bilaterally in distal segments during the initiation of a wave (posterior and anterior, respectively); however, a wave did not follow after 23 ± 24% of synchronous activity in posterior segments and 12 ± 15% in anterior segments (Fig. 1, *G* and *H*; Supplemental Movie S3; *N* = 10).

#### Comparing optical and electrophysiological recordings.

**Fig. 2.**
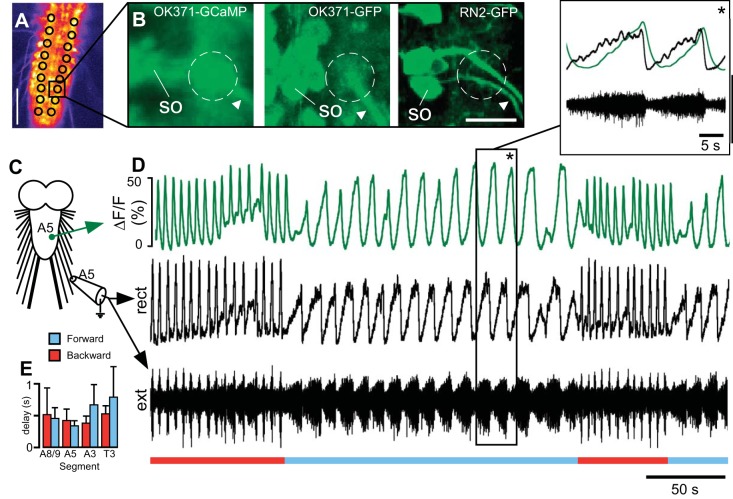
Comparison of Ca^2+^ signals with suction electrode recordings from common nerve roots (CNRs). *A*: single frame of Ca^2+^ signal showing regions of interest (ROIs) used. Scale bar, 100 μm. *B*: enlarged view of black square in *A*, showing position of ROI (dashed circle) for in vivo Ca^2+^ signal (*left*, OK371-GCaMP) compared with confocal stacks of fixed preparations showing glutamatergic neurons (*center*, OK371-GFP) and 2 motor neurons (*right*, RN2-GFP); data from 3 different animals. A single soma (SO) and CNR (arrowhead) are labeled in each image. Scale bar, 15 μm. *C*: diagram of recording site for imaging and suction electrode recording. *D*: simultaneous optical [% change in fluorescence from baseline (ΔF/F)] and extracellular suction electrode recording (ext) from CNR on right side of 5th abdominal segment. Nerve signals were rectified and filtered with a moving average filter with a time constant of 0.1 s for comparison (rect). Forward (blue line) and backward (red line) waves are indicated. *Inset*, expanded view of region with black box and asterisk. Ca^2+^ signal (green trace, *top*) and rectified, low-pass electrode signal (black trace, *top*) are overlaid, with unfiltered electrode signal (black trace, *bottom*). *E*: delay between peak of motor activity in nerve and Ca^2+^ signal in several segments for forward and backward waves (4 segments in 7 animals, ANOVA, 1 segment per animal).

We measured fluorescence values from a region of interest (ROI) in which motor neuron axons come into close proximity with each other at the base of the CNR in each ganglionic hemisegment (Fig. 2, *A* and *B*). This allowed the combined signal from all motor neurons to be measured. We compared optical signals from this region to suction electrode recordings from CNRs obtained simultaneously (Fig. 2, *C* and *D*). Spiking activity in the CNR was always coupled to the Ca^2+^ signal (Fig. 2*D*), and the signals were highly coherent during both forward and backward Ca^2+^ waves (both 99 ± 1% magnitude; forward *N* = 6, backward *N* = 4). The maximum fluorescence was delayed by 0.52 ± 0.11 s relative to the peak of activity in nerve recordings (Fig. 2, *D* and *E*; *N* = 7, average of forward and backward waves in 4 segments, 1 segment per animal). Delays within this range are expected because of the kinetics of the indicator used (Tian et al. 2009). The close correspondence between the optical signal and nerve recordings implies that the Ca^2+^ signal is a reliable indicator of motor output from each segment.

#### Quantification of motor patterns in the isolated CNS.

**Fig. 3.**
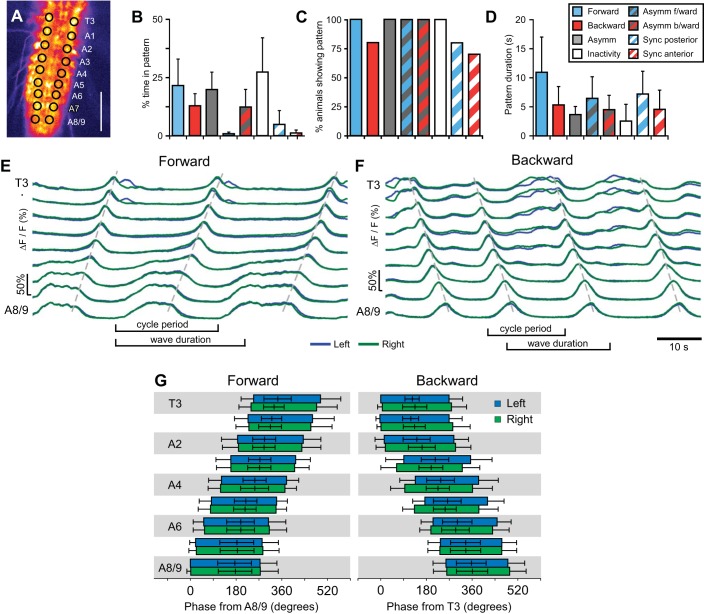
Quantification of Ca^2+^ waves. *A*: single frame of Ca^2+^ signal showing ROIs used. Scale, 100 μm. *B–D*: quantification of % of time spent in each pattern (*B*), % of animals showing each pattern (*C*), and pattern duration (*D*). *E* and *F*: Ca^2+^ signals in T3-A8/9 in the isolated CNS during forward waves (*E*) and backward waves (*F*). Signals from left (blue) and right (green) sides are overlaid. Dashed lines drawn manually over peaks for guidance. *G*: phase plot of forward (*left*) and backward (*right*) Ca^2+^ waves, showing onset, peak, and offset of activity for the left (blue) and right (green) sides. Phase is given relative to onset of activity in A8 for forward waves and T3 in backward waves. Note phases > 360°, indicating overlap of waves.

Measured from ROIs in segments T3–A8/9 (Fig. 3*A*), the CNS produced forward waves 21.4 ± 11% of the time, backward waves 12.8 ± 5.1% of the time, and bilaterally asymmetric activity 20 ± 7% of the time (Fig. 3*B*). The CNS also remained inactive for 27 ± 15% of the time (Fig. 3*B*; *N* = 10). The majority of animals showed all of these patterns (Fig. 3*C*). Forward Ca^2+^ wave duration was 10.9 ± 6.0 s, measured from the onset of fluorescence increase in A8/9 to the offset of fluorescence in T3 (Fig. 3, *D* and *E*; *N* = 10), Backward wave duration was shorter (*P* = 0.018), lasting 5.3 ± 3.1 s, measured from the onset in T3 to the offset in A8/9 (Fig. 3, *D* and *F*; *N* = 10). Both forward and backward Ca^2+^ waves frequently occurred in repetitive bouts, with a second wave commencing before the end of a first (Fig. 3, *E* and *F*); the number of waves in a bout was irregular. Within bouts, forward waves repeated with a cycle period of 8.1 ± 2.8 s (measured from start of activity in A7 in 2 successive waves; *N* = 9) whereas backward waves were more frequent (*P* < 0.001), with cycle periods of 5.8 ± 1.4 s (*N* = 6). Phase plots of segmental activation times are shown in Fig. 3*G*.

**Fig. 4.**
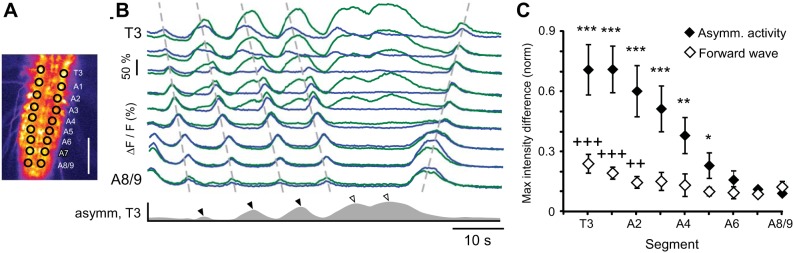
Bilaterally asymmetric Ca^2+^ signals. *A*: single frame of Ca^2+^ signal showing ROIs. Scale bar, 100 μm. *B*: Ca^2+^ signals from T3-A8/9 during bilaterally asymmetric activity. Dashed lines drawn manually over peaks for guidance. Asymmetry between the left and right side of the animal is indicated for T3 (gray trace, below). Peaks of asymmetry during backward waves (filled arrowheads) and isolated asymmetry (open arrowheads) are indicated. *C*: normalized peak difference in Ca^2+^ signal across midline for T3-A8/9 during bilaterally asymmetric activity and forward waves. Crosses show significant difference from least asymmetric segment, A7. Asterisks indicate a significantly higher L-R difference during bilaterally asymmetric activity than during forward waves (asterisks, Kruskal-Wallis 1-way ANOVA, Neuman Keuls post hoc tests). *P < 0.05, ^++^/**P < 0.01, ^+++^/***P < 0.001.

To quantify bilateral asymmetry, we calculated the absolute difference between the Ca^2+^ signal on the left and right sides of the CNS (Fig. 4) and measured the degree of asymmetry during the peak of each period of asymmetry (Fig. 4*B*). For analysis, the degree of asymmetry was normalized to the maximum peak difference in each animal. The degree of asymmetry was lowest during forward waves, so they were used as a measure of baseline asymmetry in the isolated CNS. During these waves, left-right asymmetry was significantly higher in anterior segments T3–A2 (Fig. 4*C*; *N* = 10, at least 5 waves per animal). During bilaterally asymmetric Ca^2+^ changes that were not coupled to a forward or backward wave (Fig. 4*B*), a large left-right asymmetry was observed in anterior segments and was only significantly higher than for forward waves in segments T3–A5 (Fig. 4*C*; *N* = 5, at least 3 per animal). This indicates that bilateral asymmetry in the isolated CNS is restricted to anterior segments.

#### An intrasegmental phase relationship between motor neurons during crawling also occurs in the isolated CNS.

**Fig. 5.**
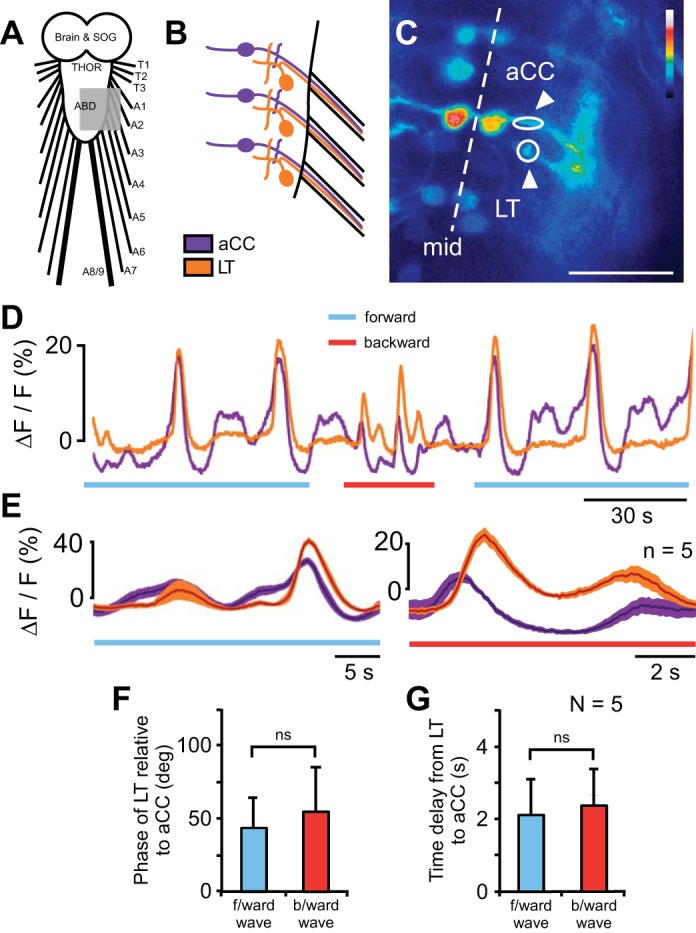
Intrasegmental coordination between motor neurons. *A*: schematic diagram of CNS; gray area represents field imaged. *B*: magnified schematic diagram of motor neurons imaged; aCC shown in purple and 1 of a pool of LT MNs in orange. *C*: single frame of Ca^2+^ signal in the nerve cord of an RRAF-GCaMP; BAR-GCaMP animal. ROIs indicated by white arrowheads. Scale bars, 25 μm. *D*: Ca^2+^ signal in aCC (purple) and LT MN neurites (orange) during forward and backward Ca^2+^ waves. *E*: average waveforms in a single animal for forward (*left*) and backward (*right*) waves; average of 5 waves. *F* and *G*: phase (*F*) and time delay (*G*) between activity in aCC and LT MNs for forward and backward waves. ns, Not significant.

In crawling *Drosophila* larvae, dorsal longitudinal (DL) muscles within a given segment contract before transverse (T) muscles during both forward and backward locomotion (Heckscher et al. 2012). To examine whether this relationship was also present in the isolated CNS, we simultaneously imaged Ca^2+^ fluorescence in the neurites of motor neuron aCC, which innervates DL muscles, and a pool of LT MNs, which innervate T muscles (Fig. 5, *A–C*; Supplemental Movie S4).

Both the LT MNs and aCC were active during forward and backward Ca^2+^ waves (Fig. 5*D*; Supplemental Movie S4). Ca^2+^ signals were complex, with variable baselines and irregular fluorescence changes (Fig. 5, *D* and *E*). Nevertheless, the waveforms of the Ca^2+^ signals in the LT MNs and aCC were significantly coherent, and phase relationships between them were derived (Fig. 5*F*). During forward waves the LT MN activity was delayed by 42 ± 22° relative to aCC (min 11°, max 94°; *n* = 22 bouts in 5 animals), and during backward waves it was delayed by 55 ± 31° relative to aCC (min −6.3°, max 110°; *n* = 14 bouts in 5 animals). The phase delay was not significantly different between forward and backward waves (*P* = 0.23, *t*-test). Expressed as time (Fig. 5*G*), the delay between aCC and LT was 2.1 ± 1.7 s for forward waves (range: 0.34–7.9 s) and 2.4 ± 1.6 s for backward waves (range: −0.2–6.52 s). Therefore, the order of recruitment of motor neuron groups in the isolated CNS was the same as the order of recruitment of muscle groups during crawling.

#### Which regions of the larval CNS are required for generating motor patterns?

**Fig. 6.**
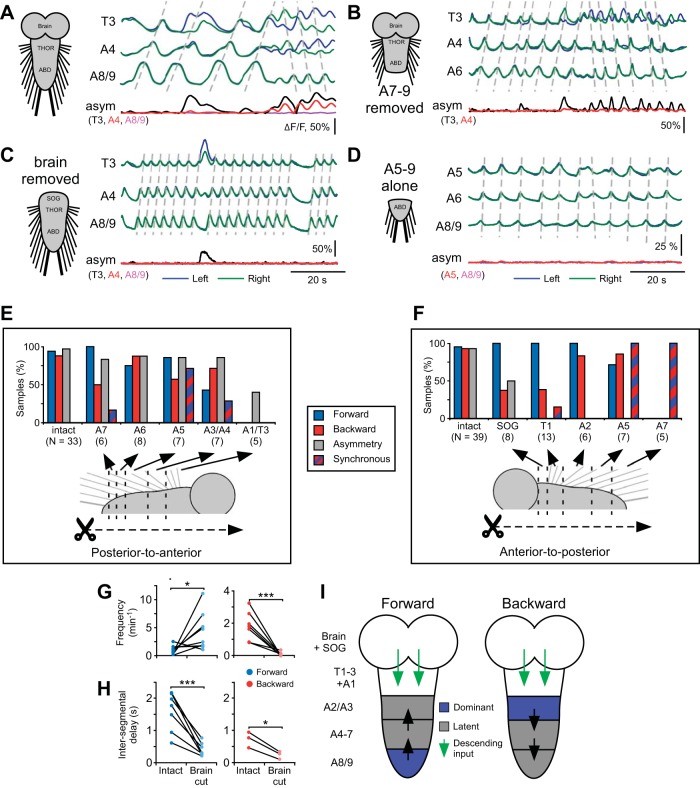
Effect of ablation of CNS segments on motor pattern generation. *A–D*: Ca^2+^ signals in 3 segments in intact CNS (*A*) and after the ablations indicated on *left* of each panel (*B–D*). Signals from left (blue) and right (green) sides are overlaid. Dashed lines drawn manually over peaks for guidance. Bilateral asymmetry of fluorescence (asym) is shown for segments indicated below. *E* and *F*: proportion of samples displaying patterns after sequential segmental ablation in a posterior-to-anterior direction (*E*) or an anterior-to-posterior direction (*F*). Sample sizes shown in parentheses. *G* and *H*: frequency (*G*) and intersegmental propagation time (*H*) of forward and backward waves before and after ablation of the brain. *I*: model of the role of segments in pattern generation for forward (*left*) and backward (*right*) waves. Segments normally first active in a wave (dominant) are indicated in blue. Segments not normally first active but still capable of initiating a wave (latent) are indicated in gray. Segments not able to initiate a wave are indicated in white. Descending signals from the brain are indicated by green arrows. Direction of wave propagation is indicated by black arrows. *G*: *P < 0.05, ***P < 0.001, ANOVA, Tukey-Kramer post hoc test. *H*: *P < 0.05, ***P < 0.001, unpaired *t*-test.

To determine which regions of the CNS were necessary for generating forward and backward Ca^2+^ waves, we imaged the preparations before (Fig. 6*A*) and after (Fig. 6, *B–D*) surgical ablation of defined CNS regions.

In the intact isolated CNS, forward waves initiated in posterior segments A7–A8/9. To test whether these regions were necessary to initiate forward waves, we ablated segments progressively in a posterior-to-anterior direction (Fig. 6, *B* and *E*; 1 ablation per preparation). After removal of segments A7 and A8/9 100% of preparations still produced forward waves (Fig. 6, *B* and “A6” in *E*; *N* = 6), and after progressively more anterior ablations the CNS still produced forward waves until cuts were targeted to A2/3, at which the CNS no longer produced either forward or backward waves (“A1/T3” in Fig. 6*E*).

Backward Ca^2+^ waves normally initiated in thoracic segments, and to test whether these regions were necessary to initiate backward waves we ablated segments progressively in an anterior-to-posterior direction (Fig. 6, *C*, *D*, and *F*). After the removal of the brain, SOG, thoracic segments, and the first abdominal segment, backward waves were still produced (“A2” in Fig. 6*F*; *N* = 8). After progressively more posterior ablations, such as when segments anterior to A5 were removed (Fig. 6, *D* and “A5” in *F*), the remaining segments were able to produce both backward and forward waves (*N* = 7). Bilaterally symmetric rhythmic bouts also occurred synchronously across several segments, during which forward and backward waves could not be distinguished (synchronous in Fig. 6*F*). After ablation of all segments anterior to A7, the remaining segments produced repetitive Ca^2+^ signals, although backward and forward waves were not distinguishable (“A7” in Fig. 6*F*; *N* = 5). Together, these results suggest that although waves normally begin in distal segments (Fig. 6*I*), those segments are not necessary to initiate waves; waves were still produced if any segment from around A2/3 to A8/9 was left intact (summarized in Fig. 6*I*).

We also investigated the production of bilateral asymmetric activity after surgical manipulation. When segments were successively ablated in a posterior-to-anterior direction (Fig. 6, *B* and *E*), the remaining CNS still produced asymmetric activity even after the removal of all segments posterior of A1, after which neither forward nor backward waves were produced (“A1/T3” in Fig. 6*E*; 40% of preparations, *N* = 5). However, when segments were ablated in an anterior-to-posterior direction, after the brain was removed the occurrence of bilateral asymmetry was greatly reduced (Fig. 6, *C* and “SOG” in *F*), from 2.2 ± 1.0 to 0.13 ± 0.15 bilaterally asymmetric episodes per minute (*P* < 0.001, *N* = 8). After removal of the brain and SOG, bilateral asymmetry was completely abolished (“T1” in Fig. 6*F*; *N* = 8). This implies that descending input from the brain and SOG (Fig. 6*I*) is involved in producing bilateral asymmetry during fictive crawling.

After removal of the brain, there was also an increase in forward wave frequency, from 0.9 ± 0.9 to 5.3 ± 3.6 waves/min (Fig. 6*G*; *P* = 0.02, *N* = 8), as well as a decrease in backward wave frequency, from 2.0 ± 0.9 to 0.07 ± 0.11 waves/min (Fig. 6*G*; *P* < 0.001, *N* = 8). In addition, there was a decrease in intersegmental delay between abdominal segments during both forward waves, reduced from 1.6 ± 0.4 to 0.4 ± 0.2 s/segment, and backward waves, reduced from 0.7 ± 0.2 to 0.2 ± 0.1 s/segment (Fig. 6*H*; forward waves: *P* = 0.001, *N* = 8; backward waves: *P* = 0.02, *N* = 3). This implies that descending input from the brain also has a role in setting the frequency and propagation rate of both forward and backward waves.

#### Comparison of fictive patterns and behavior.

**Fig. 7.**
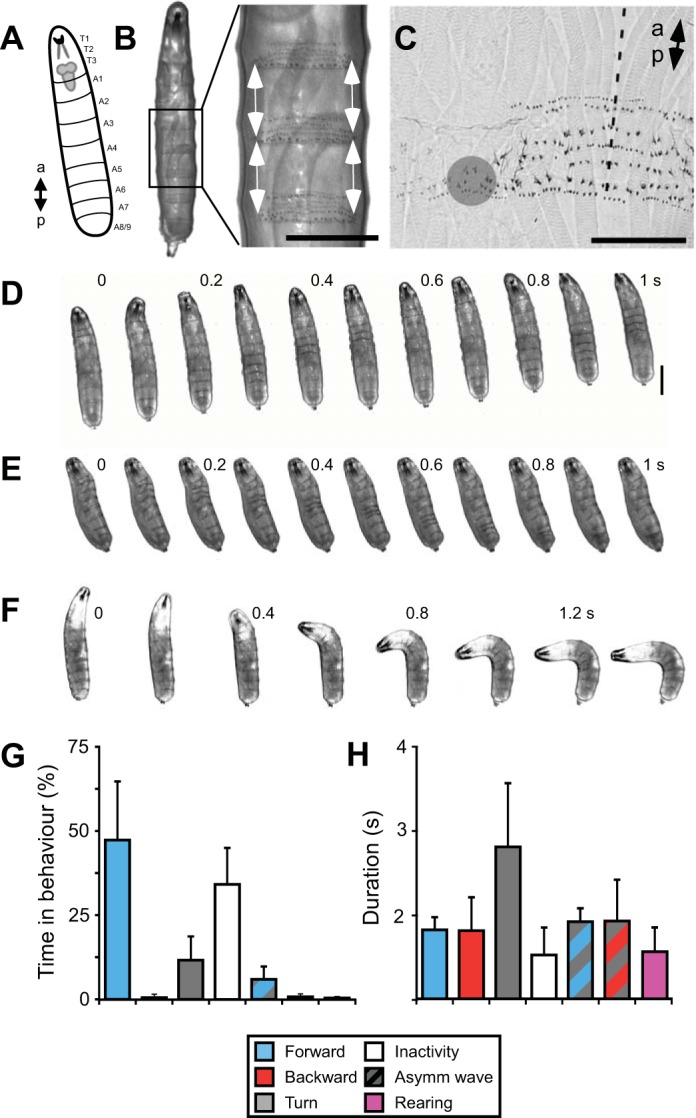
Kinematics of larval locomotion. *A*: diagram of larva with position of CNS shown. *B*: ventral view of 3rd instar larva. The contraction of each body segment was measured by manually tracking the distance between adjacent denticle bands (indicated with arrows). Scale bar, 0.5 mm. *C*: magnified view of denticle bands and tracking point (gray circle) in a fixed preparation. Dashed line indicates ventral midline. Scale bar, 0.2 mm. *D–F*: ventral view of larva during forward crawling (*D*), backward crawling (*E*), and turning (*F*). Scale bar, 1 mm. Numbers indicate time in seconds. *G* and *H*: quantification of frequency of occurrence (*G*) and duration (*H*) of each behavior.

For comparison with the patterns of motor neuron activity in the isolated CNS, we measured the patterns of segmental contraction during crawling from the movements of the denticle bands (Fig. 7, *A–C*; Supplemental Movie S5). Forward crawling involved a posterior-to-anterior propagation of segmental contractions (Fig. 7*D*), whereas backward locomotion involved an anterior-to-posterior propagation (Fig. 7*E*). Bilaterally asymmetric movements also occurred, especially during turning behavior (Fig. 7*F*; Supplemental Movie S6). Bilateral asymmetry was more frequently associated with backward waves, occurring in 63 ± 21% of waves, than with forward waves, occurring in 11 ± 7% of waves (*P* = 0.002, *N* = 10). Larvae also reared off the substrate (not shown), which has no clear analog in the isolated CNS. However, the isolated CNS showed synchronous activation of distal segments that were not followed by waves, which the larva did not. Any relationship between these two patterns is unclear.

The percentage of time spent in each pattern during behavior was different from its putative fictive equivalent in the isolated CNS (Fig. 7*G*; compare with Fig. 3*D*); behaving larvae spent 47 ± 17% of the time producing forward waves, ∼2 times more than the isolated CNS (*N* = 10, *P* = 0.003 same as isolated), whereas they spent <0.6% of the time producing backwards waves, ∼25 times less than the isolated CNS (*P* < 0.001 same as isolated). However, series of backward waves could be induced by tying dental floss around thoracic segments. Individual forward waves during behavior were ∼10 times faster than forward Ca^2+^ waves, with wave durations of 1.0 ± 0.2 s, measured from the onset of contraction in A7 to the offset of contraction in A1 (Fig. 7*H*; *P* = 0.002, *N* = 10). Backward waves during behavior were ∼10 times faster than backward Ca^2+^ waves, with wave durations of 1.0 ± 0.5 s, measured from onset in A1 to offset in A7 (Fig. 7*H*; *P* = 0.003, *N* = 10). Within bouts of continuous forward and backward crawling, cycle periods were also shorter in intact animals, with forward waves repeating at 0.98 ± 0.26-s intervals, approximately eight times shorter than forward Ca^2+^ waves (*P* < 0.001, *N* = 11), and backward waves repeating at 1.79 ± 0.56-s intervals, approximately three times shorter than backward Ca^2+^ waves (*P* < 0.001, *N* = 13).

**Fig. 8.**
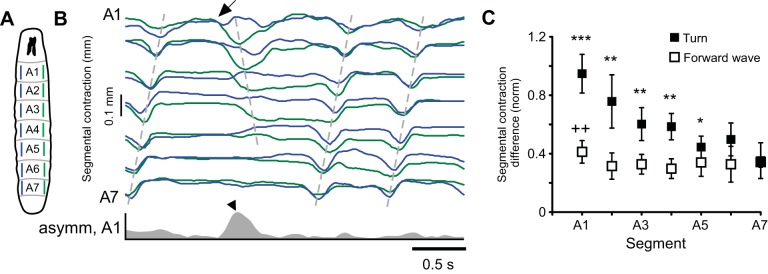
Bilateral asymmetry during turning behavior. *A*: diagram of larva with measured segments indicated. *B*: representative trace of segmental contraction during a turn. Dashed lines drawn manually over peaks for guidance. Commencement of turn indicated with arrow. Asymmetry between the left and right sides of the animal is shown for A1 (gray trace, *bottom*). Peak asymmetry during turn is indicated (arrowhead). *C*: peak bilateral asymmetry for forward waves and turns. Crosses indicate significant difference from least asymmetric segment, A4, during forward waves. Asterisks indicate segments showing significantly greater asymmetry during turns than during forward waves (Kruskal-Wallis 1-way ANOVA, Neuman Keuls, post hoc tests). *P < 0.05, ^++^/**P < 0.01, ***P < 0.001.

Similarities were found between bilateral asymmetric activity in the CNS and turning behavior (Fig. 8). They occurred equally frequently: 3.0 ± 1.6 times per minute for intact animals and 3.8 ± 2.3 times per minute in the isolated CNS (*P* = 1.0). We used the peak absolute segmental contraction difference between the left and right sides to quantify this asymmetry (Fig. 8*B*). Similar to forward Ca^2+^ waves in the CNS, the peak degree of bilateral asymmetry was low during forward waves and was significantly higher in anterior segments (Fig. 8*C*). This was used as a measure of baseline asymmetry and compared to the peak difference in contraction during turning behavior. Segments A1–A5 showed greater asymmetry during turning behavior than during forward crawling (Fig. 8*C*). This restriction in asymmetry to anterior segments was also observed during bilateral asymmetry in the isolated CNS, when asymmetry occurred in T3–A5 (Fig. 4*C*). These similarities imply that bilateral asymmetry in the isolated CNS could be a fictive equivalent of turning in the behaving animal.

#### Intersegmental phase relationships in the isolated CNS resemble those observed in intact animals.

**Fig. 9.**
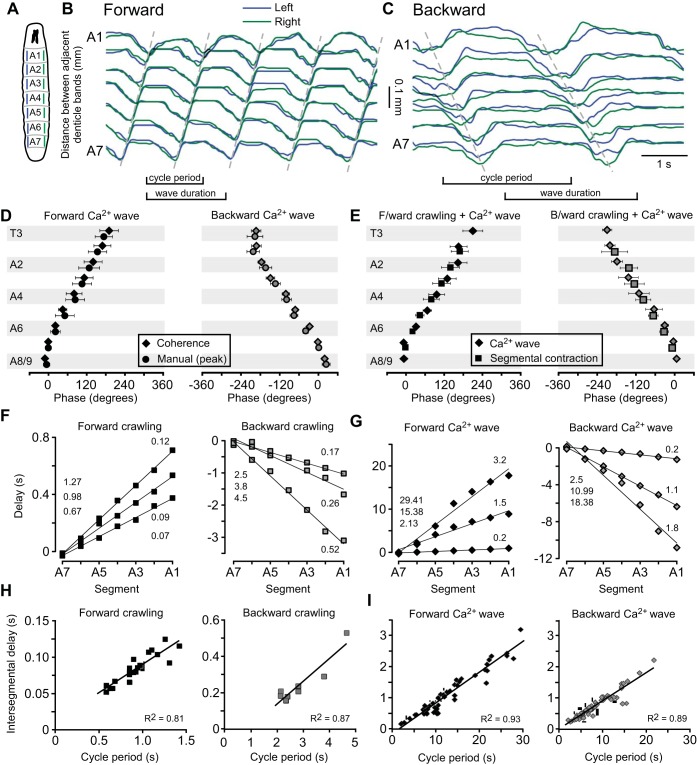
Quantification of segmental contraction during crawling and comparison with Ca^2+^ waves. *A*: diagram of larva with measured segments indicated. *B* and *C*: representative traces of segmental contraction in segments A1–A7 during forward crawling (*B*) and backward crawling (*C*). Dashed lines drawn manually over peaks for guidance. *D*: phase data from Ca^2+^ waves analyzed by coherence analysis and the peak of waves measured manually. No significant differences were found for any segment between the 2 methods during forward (*left*) and backward (*right*) waves. *E*: phase of segmental activation, calculated by coherence analysis, for Ca^2+^ waves (diamonds) and crawling behavior (squares) for forward (*left*) and backward (*right*) waves. No significant differences were found in phase delay in any segment between Ca^2+^ waves and segmental contraction. *F* and *G*: linear scaling of segmental activation time as a function of segment number for crawling behavior (*F*) and Ca^2+^ waves (*G*) during forward (*left*) and backward (*right*) waves. Cycle period is indicated on *left* and slope on *right* of each panel. The slope was used as a measure of intersegmental delay. *H* and *I*: linear scaling of intersegmental delay with cycle period for crawling behavior (*H*) and Ca^2+^ waves (*I*) for forward (*left*) and backward (*right*) waves. Phase values were measured relative to A7 on left side for all data.

We analyzed the phase relationships between body segments based on the patterns of contraction during forward and backward crawling (Fig. 9, *A–C*) and compared them with the phase of activation of ganglionic segments during Ca^2+^ waves in the isolated CNS (Fig. 3). To calculate these phase relationships, we used coherence analysis (see materials and methods; Cacciatore et al. 1999; Rehm et al. 2008). Measuring phase with this method yielded values similar to manual analysis for both forward and backward Ca^2+^ waves (Fig. 9*D*; *P* = 0.28 and *P* = 0.85, respectively, 2-way ANOVA). Within a forward wave, average phase angles for segments A1–A7 in the isolated CNS were statistically indistinguishable from corresponding phase angles measured for segmental contraction during forward crawling in intact larvae (Fig. 9*E*, *left*; *P* = 0.76, 2-way ANOVA). This was also the case for backward Ca^2+^ waves when compared with induced bouts of backward crawling (Fig. 9*E*, *right*; *P* = 0.40, 2-way ANOVA).

#### Intersegmental delays scale linearly with cycle period in isolated CNS and intact larvae.

To investigate how phase relationships were maintained within the wide range of cycle periods observed in both the isolated CNS and behaving larva, we analyzed the scaling of intersegmental delay with cycle period. Time delays were calculated by multiplying cycle period by the phase angles derived from coherence analysis. The interval between contractions in adjacent segments did not vary along the body axis from A1 to A7; the time of segmental contractions scaled linearly as a function of segment number for both forward and backward waves during crawling (Fig. 9*F*; forward *R*^2^ = 0.98 ± 0.02, backward *R*^2^ = 0.98 ± 0.01). Ca^2+^ waves in the isolated CNS also showed a linear scaling of activity in A1–A7 as a function of segment number (Fig. 9*G*; forward *R*^2^ = 0.97 ± 0.02, backward *R*^2^ = 0.95 ± 0.05). We therefore used the slope of these regression lines as a measure of intersegmental delay and analyzed whether it scaled across a range of cycle periods (cf. Cacciatore et al. 2000).

Intersegmental delay scaled linearly with cycle period during forward and backward crawling behavior (Fig. 9*H*; forward *R*^2^ = 0.81, backward *R*^2^ = 0.87) and for the isolated CNS for both forward and backward Ca^2+^ waves (Fig. 9*I*; forward *R*^2^ = 0.93, intact *R*^2^ = 0.81). This implies that as cycle period increases, so does the intersegmental delay. Furthermore, the slope of scaling was similar in the intact larvae and isolated CNS for both forward waves (intact 0.08 ± 0.04, isolated 0.10 ± 0.03; *P* = 0.90, *t*-test) and backward waves (intact 0.12 ± 0.06, isolated 0.09 ± 0.04; *P* = 0.90, *t*-test). This linear scaling appears to be a core feature of the larval locomotor CPG network that is reflected in larval crawling behavior.

## DISCUSSION

The isolated CNS of *Drosophila* larvae spontaneously produced three distinct motor patterns, measured from Ca^2+^ signals in glutamatergic neurons. Two were metachronal waves that progressed in a posterior-to-anterior or anterior-to-posterior direction. These waves of motor activity resembled forward and backward crawling motor patterns in the order and phase of segmental activation. A third motor pattern was bilaterally asymmetric activity, which resembled turning behavior both in the frequency of its occurrence and the restriction of asymmetry to anterior segments. All three motor patterns occurred endogenously without external stimulation.

The presence of fictive locomotor patterns, which occur without sensory feedback, has been observed in many animals (see for reviews Delcomyn 1980; Marder and Calabrese 1996; Mulloney and Smarandache 2010). However, in *Drosophila*, fictive locomotor patterns had previously been observed primarily in semi-intact preparations (Berni et al. 2012; Fox et al. 2006; Kohsaka et al. 2014; McKiernan 2013; Schaefer et al. 2010) and only recently in the isolated preparation (Berni 2015; Lemon et al. 2015). These studies also included a coarse analysis of the delays between segments. Our work advances beyond this by providing a detailed description of intersegmental coordination in isolated nerve cords and intact animals, aided by the use of coherence-based analysis; describing how this coordination scales with different cycle periods; characterizing intrasegmental phase relationships among motor neurons in the isolated CNS; and providing new insights into roles of CNS regions in generating the observed motor patterns. These advances, taken together with previous work, verify the presence of a CPG network underlying locomotion in *Drosophila* larvae and provide an essential basis for dissecting the network at a circuit level.

The intrasegmental order of motor neuron recruitment during the fictive pattern was the same as during crawling in intact larvae (Fig. 5; Heckscher et al. 2012). The coordination between motor neurons within a segment during fictive behavior has been observed in a wide range of behaviors, including flying (Wolf and Pearson 1989), singing (Schöneich and Hedwig 2012), walking (Büschges et al. 1995; Grillner and Zangger 1984; Pearson 1972), swimming (Friesen and Hocker 2001; Soffe and Roberts 1982), and mastication (see for review Marder and Bucher 2007). The persistence of the phase relationships between motor neurons during fictive behavior in larval *Drosophila* suggests that the mechanisms to maintain the coordination between muscle groups are contained within the CNS and operate without sensory feedback. However, the large variance in the delays observed suggests that sensory feedback may act to refine the phase relationship.

Fictive motor patterns are often slower than their putative equivalent patterns during behavior (Harris-Warrick and Cohen 1985; Kristan and Calabrese 1976; Wilson 1961; Zhong et al. 2012). Here, we observed wave durations that were ∼10 times shorter during crawling behavior than during Ca^2+^ waves in the isolated CNS (Fig. 2*B*, Fig. 7*B*). The influence of sensory feedback in speeding up the pattern of motor activity is supported by studies using genetic tools to selectively inactivate either sensory neurons (Caldwell et al. 2003; Hughes and Thomas 2007; Song et al. 2007; Suster and Bate 2002) or premotor neurons that receive sensory input (Kohsaka et al. 2014). Similarly, in semi-intact preparations of larval *Drosophila* the conduction time between segments is greatly increased (Berni et al. 2012; Fox et al. 2006; Schaefer et al. 2010) and is increased further when premotor neurons are silenced (Kohsaka et al. 2014). We have also observed more variability in the cycle period of motor activity in fictive locomotor patterns than in crawling patterns (Fig. 9). This has also been observed in other CPG networks controlling movement over terrain (Eisenhart et al. 2000; Fischer et al. 2001), suggesting that CPGs for walking and crawling appear to be heavily reliant on sensory feedback and produce irregular output when it is absent (reviewed in Marder et al. 2005).

A further difference between the fictive patterns and behavior was the likelihood to produce forward and backward waves; the isolated CNS spent roughly an equal proportion of time producing fictive forward and backward waves (Fig. 3*B*), whereas crawling larvae showed a heavy bias toward forward crawling (Fig. 7*G*). The bias to produce forward waves in intact larvae is lost when genetic manipulations are used to abolish sensory feedback (Hughes and Thomas 2007; Suster and Bate 2002) and is also lost in semi-intact preparations (Berni et al. 2012; Fox et al. 2006; Schaefer et al. 2010). Here, we could bias intact larvae toward producing backward waves by constricting thoracic body segments. Together these data suggest that sensory stimuli influence the direction of larval locomotion.

### 

#### Role of CNS regions in producing fictive patterns.

The ability of the abdominal and thoracic ganglia of insects to generate locomotor behavior without the brain has long been appreciated (Roeder 1937; Roeder et al. 1960) but has only recently been shown in larval *Drosophila* (Berni et al. 2012). In experiments here, both forward and backward waves still occurred when the brain was removed. The occurrence of locomotor patterns after removal of the brain has also been observed in the leech (Mullins et al. 2012), caterpillar (Dominick and Truman 1985), locust (Kien 1983), and cockroach (Ridgel et al. 2007), among other invertebrate species. However, the degree of dependence on the brain seems to differ between animal groups, as in highly cephalized vertebrates the nervous system appears to rely on the brain for the initiation of locomotor behavior (Soffe et al. 2009; Whelan 1996).

The brain and SOG have previously been implicated in affecting the likelihood to produce particular patterns of locomotor behavior in several species (Dominick and Truman 1985; Kien 1983; Kien and Altman 1984; Mullins et al. 2012). We have noticed an increase in the frequency of forward waves but decrease in backward waves once the brain was removed (Fig. 6*G*). Accompanying this was a decrease in the frequency of bilaterally asymmetric activity. The brain and SOG have previously been implicated in turning behavior in the cockroach (Ridgel et al. 2007; Ye and Comer 1996) and cricket (Zorović and Hedwig 2013) and also recently in *Drosophila* larvae (Tastekin et al. 2015). The complete abolition of turning behavior once the SOG was removed conflicts with recent experiments in which the brain and SOG have been silenced with genetic tools when turning behavior still occurred (Berni 2015; Berni et al. 2012). It is possible that neurons important for generating bilateral asymmetry have somata in the VNC and project anteriorly to the SOG or brain. These may not be silenced by genetic manipulation but were severed during our surgical manipulation. Further work is required to clarify the position, projection patterns, and roles of neurons regulating bilateral asymmetry.

The role of the thoracic and abdominal ganglia in generating locomotor activity has also been studied here. In several species, single ganglia have been shown to produce cyclical motor patterns in isolation, but coordination of their activity through intersegmental connections is necessary to produce a pattern relevant to behavior (locusts: Lewis et al. 1973; Ramirez and Pearson 1989; crayfish: Murchison et al. 1993; leeches: Hocker et al. 2000; Puhl and Mesce 2008) The small size of the larval VNC and the fused nature of the ganglia prevented us from testing the ability of single isolated segments to produce motor patterns. However, through sequential surgical ablation of segments in either a posterior-to-anterior or anterior-to-posterior direction, we propose that any segment from A2/3 to A8/9 is able to initiate both forward and backward waves (summarized in Fig. 6*I*). This suggests that the network components underlying pattern initiation are distributed across multiple segments within the larval locomotor network.

#### Scaling of intersegmental delay.

A linear scaling of intersegmental delay with cycle period occurred in segments A1–A7 for both forward and backward waves during crawling behavior and fictive locomotion (Fig. 9). Linear scaling of the time delay of components of locomotor patterns has been observed in a wide range of animals during behavior (*Drosophila*: Heckscher et al. 2012; caterpillars: Johnston and Levine 1996; cockroaches: Pearson and Iles 1970; leeches: Stern-Tomlinson et al. 1986; cats: Goslow et al. 1973; humans: Nilsson et al. 1985). Similar scaling has also been observed in preparations producing fictive motor patterns (crustaceans: Bucher et al. 2005; caterpillars: Johnston and Levine 1996; lampreys: Grillner et al. 1991; dogfish: Grillner 1973). The linear scaling of intersegmental delay with cycle period during fictive crawling in *Drosophila* suggests that neuronal mechanisms to maintain intersegmental coordination across a variety of network speeds are contained within the CNS and operate without sensory feedback.

#### Role of Ca^2+^ in fictive patterns.

In this study, we have used the Ca^2+^ influx during fictive locomotion as an indicator of motor activity within each segment, based on simultaneously recorded Ca^2+^ signals and spike activity recorded from nerve roots (Fig. 2). However, the observed increase in cytosolic Ca^2+^ in the neurons has functional significance, as Ca^2+^ will likely have a range of effects (Berridge et al. 2000). The role of Ca^2+^ in shaping the intrinsic properties of neurons during locomotion in *Drosophila* has been studied previously through the manipulation of Ca^2+^ channels in motor neurons (Worrell and Levine 2008) and Ca^2+^-sensitive K^+^ channels in a group of interneurons (McKiernan 2013). Both studies implicate Ca^2+^ as an intracellular negative feedback signal that triggers activation of K^+^ channels, which in turn facilitate a reduction in neuronal excitability.

#### Limitations of imaging techniques.

The imaging techniques used here provide new opportunities for studying the *Drosophila* larval locomotor network but also have inherent limitations. For example, the identification of action potentials with Ca^2+^ indicators is difficult, because the relationship between intracellular Ca^2+^ and spiking activity is often complex and nonlinear, especially at high spiking rates (Franconville et al. 2011; Helmchen et al. 1996; Tian et al. 2012). Furthermore, inhibitory signals can be difficult to measure (Haynes et al. 2015). Calcium indicators also have inherent buffering capacities, which further contribute to the nonlinearities between membrane potential and Ca^2+^ signals (Helmchen et al. 1996; Tian et al. 2012). Here we report a delay of ∼0.5 s between the peaks of the electrophysiological and Ca^2+^ signals (Fig. 2), which is within the range expected from the indicator used (Tian et al. 2009). New generations of indicators are being produced to overcome these limitations, with improved signal-to-noise ratio and calcium-binding affinities (Chen et al. 2013; Sun et al. 2013). However, the use of Ca^2+^ indicators to monitor spiking activity will always be limited by the nonlinear relationship between Ca^2+^ and membrane potential. Optical voltage sensors could be used to overcome this, although their utility is currently limited by low signal-to-noise ratio (Barnett et al. 2012; Cao et al. 2013; Mutoh et al. 2011). Alternatively, neurons identified in imaging experiments could be targeted with traditional electrophysiological approaches (Baines and Bate 1998).

In the present study, we have imaged Ca^2+^ signals that were slow relative to the time constants of the indicator used. As a result, the inherent limitations should not affect the interpretation of our data. However, tracking rhythmic activity at fast timescales, within the decay time constant of the indicator, may prove difficult in future studies. However, the development of new generations of GCaMP variants with shorter decay constants should help to overcome these difficulties (Chen et al. 2013; Sun et al. 2013).

#### Outlook for future work.

In *Drosophila* larvae, the use of genetically encoded Ca^2+^ indicators like GCaMP3 provides an opportunity to image and analyze the coordination of fictive locomotor behaviors across large regions of the CNS simultaneously. Here we examined the output of larval motor neurons, but with a similar approach the expression of Ca^2+^ indicators could be targeted to subsets of interneurons or sensory neurons. Building on the present work, this should provide the opportunity to uncover the mechanisms underlying the generation and coordination of locomotion in *Drosophila* at the level of individual interneurons, which should provide further insights into the fundamental mechanisms of locomotor pattern generation.

## GRANTS

S. R. Pulver was supported by a Newton International Fellowship (Royal Society) and a Junior Fellowship (Janelia Research Campus, Howard Hughes Medical Institute). T. G. Bayley was supported by a Medical Research Council (UK) PhD grant. J. Berni was supported by a Henry Dale Fellowship (Royal Society and Wellcome Trust). M. Bate was supported by the Isaac Newton Trust.

## DISCLOSURES

No conflicts of interest, financial or otherwise, are declared by the author(s).

## AUTHOR CONTRIBUTIONS

Author contributions: S.R.P., T.G.B., A.L.T., M.B., and B.H. conception and design of research; S.R.P., T.G.B., and J.B. performed experiments; S.R.P., T.G.B., and A.L.T. analyzed data; S.R.P., T.G.B., A.L.T., and B.H. interpreted results of experiments; S.R.P. and T.G.B. prepared figures; S.R.P., T.G.B., and B.H. drafted manuscript; S.R.P., T.G.B., A.L.T., J.B., and B.H. edited and revised manuscript; S.R.P., T.G.B., A.L.T., J.B., M.B., and B.H. approved final version of manuscript.

## Supplementary Material

Supplemental Movie 1

Supplemental Movie 2

Supplemental Movie 3

Supplemental Movie 4

Supplemental Movie 5

Supplemental Movie 6

## Footnotes

1 Supplemental Material for this article is available online at the Journal website.
